# The Effectiveness of Health Care Information Technologies: Evaluation of Trust, Security Beliefs, and Privacy as Determinants of Health Care Outcomes

**DOI:** 10.2196/jmir.9014

**Published:** 2018-04-11

**Authors:** Victoria Kisekka, Justin Scott Giboney

**Affiliations:** ^1^ Information Security and Digital Forensics School of Business University at Albany, State University of New York Albany, NY United States; ^2^ Information Technology Department Brigham Young University Provo, UT United States

**Keywords:** medical informatics, privacy, quality of health care, trust

## Abstract

**Background:**

The diffusion of health information technologies (HITs) within the health care sector continues to grow. However, there is no theory explaining how success of HITs influences patient care outcomes. With the increase in data breaches, HITs’ success now hinges on the effectiveness of data protection solutions. Still, empirical research has only addressed privacy concerns, with little regard for other factors of information assurance.

**Objective:**

The objective of this study was to study the effectiveness of HITs using the DeLone and McLean Information Systems Success Model (DMISSM). We examined the role of information assurance constructs (ie, the role of information security beliefs, privacy concerns, and trust in health information) as measures of HIT effectiveness. We also investigated the relationships between information assurance and three aspects of system success: attitude toward health information exchange (HIE), patient access to health records, and perceived patient care quality.

**Methods:**

Using structural equation modeling, we analyzed the data from a sample of 3677 cancer patients from a public dataset. We used R software (R Project for Statistical Computing) and the Lavaan package to test the hypothesized relationships.

**Results:**

Our extension of the DMISSM to health care was supported. We found that increased privacy concerns reduce the frequency of patient access to health records use, positive attitudes toward HIE, and perceptions of patient care quality. Also, belief in the effectiveness of information security increases the frequency of patient access to health records and positive attitude toward HIE. Trust in health information had a positive association with attitudes toward HIE and perceived patient care quality. Trust in health information had no direct effect on patient access to health records; however, it had an indirect relationship through privacy concerns.

**Conclusions:**

Trust in health information and belief in the effectiveness of information security safeguards increases perceptions of patient care quality. Privacy concerns reduce patients’ frequency of accessing health records, patients’ positive attitudes toward HIE exchange, and overall perceived patient care quality. Health care organizations are encouraged to implement security safeguards to increase trust, the frequency of health record use, and reduce privacy concerns, consequently increasing patient care quality.

## Introduction

### Background

Today, the health care industry primarily relies on health information technologies (HITs) such as electronic medical record (EMR) systems, patient health record (PHR) systems, and technical devices to deliver patient care services. Despite the continued diffusion of HITs within the health care sector, there is no theory explaining how HIT success influences perceived patient care quality.

Substantial strides have been made to study the success of HITs and their impact on patient care outcomes such as care quality, patient satisfaction, patient empowerment, and increased likelihood of adherence to medications [1-5]. But, with the increase in data breaches and privacy concerns [6], the success of HITs is now also contingent on how well the privacy of patient medical data is secured. There is a scarcity of empirical research evaluating the success of HITs from the perspective of information assurance. *Information assurance* is the protection of information and information systems, the detection of threats, and the reaction to threats [7]. Existing research has not answered the question: how does the success of the information assurance attributes of HITs influence perceived patient care quality?

Existing work misses 3 critical components of information assurance namely—information security beliefs, privacy concerns, and trust in health information. *Information security beliefs* are the perception of the user that data provided to the organization will be accurate and available. *Privacy concerns* are the perceived lack of confidentiality of personal information provided to the organization. Trust is the perception of the user that health information provided by the organization is reliable [8]. From an organization standpoint, organizations increase security beliefs by enacting security and privacy controls, undertaking tasks that ensure data accuracy and availability, and developing controls to protect the confidentiality of user data [8]. This research advances our understanding of the role of information security beliefs, privacy concerns, and trust in health information in a success model of a health care system. We extend existing research by going beyond the influence of privacy concerns. We included 2 new determinants of patient care quality—information security beliefs and trust in health information.

The objective of this research is threefold; first, we seek to examine the role of information assurance constructs (ie, information security beliefs, privacy concerns, and trust in health information) as measures of HIT effectiveness. Second, we seek to empirically investigate the critical yet unknown relationship between information assurance constructs and three aspects of system success: attitude toward health information exchange (HIE), patient access to health records, and perceived patient care quality. Third, our research extends current literature by extending the DeLone and McLean Information Systems Success Model (DMISSM) to the information assurance area in a health care context. We added a new variable to the model namely—attitude toward HIE.

### Prior Literature

Ever since the call for improving patient care quality outcomes by Institute of Medicine [9]; a growing stream of health care research has explored the relationship between HIT use and numerous aspects of patient satisfaction with the health care organization. The correlates of HIT use include improved care coordination, enhanced communication between providers and patients, and increased effectiveness in various measures of quality outcomes and provider performance [10-16]. Han et al [12] showed that meeting the objectives of HIT use resulted in increased patient adherence to recommended diabetes tests and reduced hospital utilization. Similar findings related to better medication management and adherence was reported in recent studies [17,18].

As health care is a service, it is important to understand users’ perceptions of HIT and service quality. Setia et al [19] theoretically and empirically demonstrate the link between information quality and service capabilities and performance. Their findings inform us that improving information quality enhances the effectiveness of service quality efforts.

A major perception of an HIT by users is information assurance. Brown et al [20] explain the trade-off between information privacy controls and patients’ access to electronic health records. They show that obtaining an optimal balance between information privacy controls and access to patients’ information primarily requires a clear understanding of the patient. Efforts for optimizing patient care quality outcomes demand health care providers to accurately identify patients’ preferences for privacy to determine the acceptable levels of access to sensitive health information without violating patients’ privacy. Brown et al’s [20] work also underscores the importance of privacy controls in achieving positive patient care outcomes.

In the context of privacy concerns, the existing literature suggests that privacy concerns influence not only patients’ perceptions of patient care quality but also behavioral intentions of HIT usage [21-25]. These findings suggest that patient satisfaction and confidence in using provider-managed technologies such as EMR and PHR technologies and nonprovider-managed HITs such as health social networks and other Web-based health information resources are obtained through strong perceptions of the effectiveness of security and privacy controls [26]. In fact, patients often express a preference for security features in Web-based patient portals [27]. We extend this body of literature by empirically testing the influence of information security beliefs, privacy concerns, and trust in health information; the role of these factors as measures of HIT effectiveness is currently not well understood. We theoretically extend existing literature using the DMISSM.

### The DeLone and McLean Information Systems Success Model in Health Care

**Figure 1:**
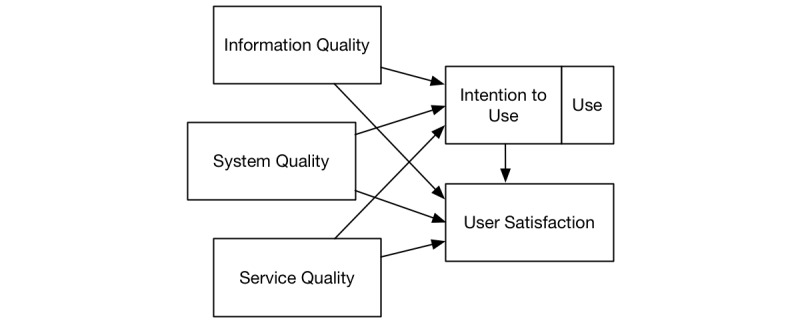
Adapted from the DeLone and McLean Information Systems Success Model.

In this study, we examined the effect of HITs on perceived health care service quality. Health care is made of HITs and services that interact together to deliver patient care [28,29]. Because this research investigates the effectiveness of HITs within health care, it is necessary to adopt a theoretical framework that can explain how the components interact and lead to perceived patient care quality. Thus, we employ the DMISSM presented in Figure 1 [30]. The DMISSM was developed to help organizations understand the benefits of information systems (IS) and how the effectiveness of IS impacts users and organizations. The model has been widely adopted to better understand IS success in different contexts [30]. We limit our discussion of DMISSM to its application in the health care discipline. The remainder of this section explains how the DMISSM has been used in previous HIT research.

As shown in Figure 1, the dimensions of success in the model are information quality, system quality, service quality, system use or intention to use, and user satisfaction [30,31]. In the context of patient safety, *information quality* refers to the completeness, relevance, accuracy, and timeliness of medical information; *system quality* refers to the usability, compatibility, reliability, and response time of the HIT; and *service quality* refers to the technical support and assurance (availability, integrity, and authenticity) of the HIT as well as the quality of service received [31]. Patients and health care providers alike, evaluate HITs in terms of information quality, system quality, and service quality [32,33], all of which are components of the DMISSM [30,31]. Beyond just HIT features, a patient’s decision to use HIT also depends on nontechnical success factors and facilitating conditions such as behavioral controls and work processes [34], HIT cost, the individual’s technical background and skill set, health conditions (eg, visual impairment), and information privacy concerns [35]. These constructs are commonly studied along with success measures of perceptions about the system such as the ease of use, usefulness, and enjoyment [36]. A large part of the purpose of an HIT is protecting and improving patient safety [31].

A significant component of HIT is how both HIT functionality and HIT use by patients influence patient care quality. Consistent with the DMISSM, HIT has 3 dimensions of quality: information, system, and service [30,37]. In turn, the perceptions of the dimensions of quality should positively influence the intention and decision to use the HIT, which consequently impacts the perceived benefit to the user. The perceived benefit to the user in our study is patient care quality. To our knowledge health care and IS research scholars have not yet made the critical link between patients’ experiences with and perceptions of HITs and patient care quality outcomes. Existing research focuses on the relationship between HIT implementation and health outcomes from a macro level (ie, are there societal benefits?) [38] but not from a micro level (ie, are there individual benefits?). Another current gap relates to studying information assurance variables as dimensions of IS success. The rife of data breaches and patients’ concern for privacy has created the need to understand HIT effectiveness from the perspective of their security. This research addresses the limitation by studying system quality and service quality in terms of the effectiveness of information security controls and information privacy safeguards.

In summary, many papers study the adoption of HITs but stop short of investigating how patients’ use of HITs affects the ultimate goal of health care organizations: quality of care (eg, [39]). Other papers have studied health outcomes in the context of patient HIT success without statistical analysis [20]. Still, others have studied the continued use of patient HIT as the ultimate dependent variable and focus solely on the technology-of-interest [21]. Papers have also studied the adoption of HIT by health care providers [40,41]. We build on this research by empirically testing the effect of patients’ perceptions of HIT quality measures and the impact of patient care quality.

### Contextualized Hypotheses

**Figure 2:**
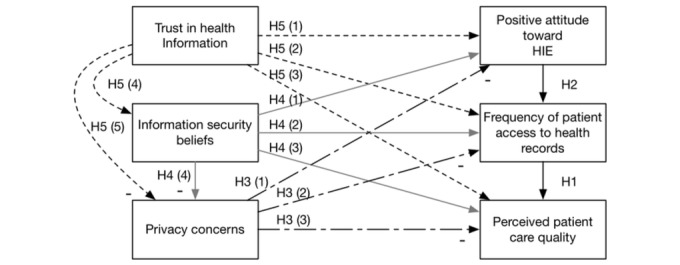
The health care system success model. HIE: health information exchange.

This research studies the technical and social elements of a health care system. In the next paragraphs, we develop hypotheses for the model (see Figure 2).

#### Patient Care Quality

The ultimate dependent variable and the raison d'être for a health care system is to increase the quality of life of patients. Closely akin to the actual quality of care is the perceived quality of care of the patient. If patients perceive to have received high-quality care, it is likely that they are actually receiving quality care. Therefore, *patient care quality* is a perception of one’s belief that he or she is receiving the best possible care from the health care system.

#### The Frequency of Patient Access to Health Records

This variable pertains to the frequency with which the patient uses the EMR to access his/her health records. Patient satisfaction has been shown to be positively associated with access to health records [42]. Under the Health Insurance Portability and Accountability Act regulation, patients have the legal right to access their medical records. Health care providers are increasingly adopting technologies, such as EMRs, to not only interact with their patients but also comply with government regulations [43]. According to DMISSM, when users engage with a system that helps them achieve their goals, they become more satisfied with the system [44]. It has also been previously established that patient access to medical records promotes communication between patients and physicians, consequently improving the quality of care that the patient receives [45,46]. Thus, we hypothesize the following:

H1. Patient access to health records increases the level of perceived patient care quality.

#### Attitude Toward Health Information Exchange

This variable pertains to the patient’s attitude toward information sharing among health care providers. A significant enabler of coordination in a health care system is the ability to share medical information electronically. Sharing of medical records facilitates timely delivery of care, which is a benefit for the patient. Sharing of medical information is, therefore, a valuable service for patients, as has been shown by researchers [47]. We argue that because of the value associated with the technical capabilities of information sharing, patients who support provider use of EMRs for information exchange may increase their own access to EMR. Patients are more accepting of HITs when they perceive the technologies to be beneficial to care delivery [48]. This acceptance includes patients’ actual use of HITs and support and endorsement of providers’ use of HITs [48]. Also, DMISSM explains that information quality and the service quality of the system increase the intention to use a system and actual use of the system [44]. Patients who desire that their health care provider exchange information using HITs are showing an intention to use EMR and participate in the health care organization as a whole. Thus, we hypothesize the following:

H2. Positive attitudes toward HIE increase the patient’s access to health records.

#### Privacy Concerns

There is an increasing amount of research related to the privacy of one’s information [49]. Privacy is especially important with regard to health care information [35]. People often desire to keep their EMR out of the public domain. Privacy concerns in the health care system are part of service quality. According to DMISSM, service quality increases intention to use and use of a system [44]. This is because users perceive the system to be more reliable. Because privacy concerns are a negative measure of service quality, as privacy concerns increase, use of EMR, positive attitude toward HIE, and ultimately, perceived quality of care will decrease. Thus, we hypothesize the following:

H3. Privacy concerns will

1. decrease patients’ positive attitudes toward HIE,
2. decrease the frequency of patient access to health records, and
3. decrease the level of perceived patient care quality.

#### Information Security Beliefs

Information security beliefs are related to privacy concerns in that both deal with the assurance (integrity, availability, and authenticity) of health information. Information security beliefs are distinct from privacy concerns because information security beliefs are the idea that the system is protecting health information, and privacy concerns are the worry that health information will not be confidential. Information security beliefs will decrease privacy concerns. Also, information security beliefs are part of service quality of the health care system. Just as privacy concerns influence intention to use and use of the system, an increase in information security beliefs will increase use of EMR, positive attitude toward HIE, and ultimately, perceived quality of care. Thus, we hypothesize:

H4. Information security beliefs will

1. increase patients’ positive attitudes toward HIE,
2. increase the frequency of patient access to health records,
3. increase the level of perceived patient care quality, and
4. decrease privacy concerns.

#### Trust in Health Information

As a component of information quality, trust in internet health information increases a user’s expected improved decision making and positive outcomes [44]. As a user’s expected improved decision making and positive outcomes increase, the likelihood that the user will continue to use the system increases. Thus, as health care system users believe that they are receiving quality information from the internet, they will continue to participate in the health care system (as explained by the DMISSM). Specifically, as trust in internet health information increases, use of EMR, positive attitudes toward HIE, and ultimately, perceived quality of care will also increase. Furthermore, the concept that health information will be tampered with will decrease. As trust in internet health information increases, a person’s information security beliefs will also increase and their privacy concerns will decrease. Thus, we hypothesize:

H5. Trust in internet health information will

1. increase patients’ positive attitudes toward HIE,
2. increase the frequency of patient access to health records, and
3. increase the level of perceived patient care quality.
4. *increase information security beliefs and*
5. *decrease privacy concerns.*

## Methods

This study employed a structural equation modeling (SEM) of the Health Information National Trends Survey (HINTS) of cancer patients to test our hypotheses [50]. SEM was chosen as the appropriate method because we are simultaneously analyzing multiple paths and multiple dependent variables, and we have a large enough sample that use of partial least squares is unnecessary. We used the HINTS 4 Cycle 4 dataset [50]. The HINTS datasets are publically available responses to surveys about health-related topics. Since the dataset is anonymized public data, there was no need for IRB approval, however, all data were kept confidential. For full details regarding the method of survey collection, see National Trends Survey [50]. The HINTS 4 Cycle 4 survey contained questions related to our phenomena of interest. Table 1 contains the questions from the survey with their corresponding construct.

The survey targeted known minority and nonminority populations. The survey targeted 1 adult per household in selected areas of the United States. In total, 3677 of 13,996 surveys (26.27%) were completed. Of the participants, 467 (12.70%) were in the age group of 18 to 34 years, 743 (20.21%) were aged between 35 and 49 years, 1220 (33.18%) were aged between 50 and 64 years, 637 (17.32%) were in the age group of 65 to 74 years, 428 (11.64%) were older than 75 years, and 182 (4.95%) did not specify. Of the participants, 2184 (59.40%) were female, 1424 (38.72%) were male, and 69 (1.88%) did not specify. Of the participants, 90 (2.45%) had completed less than 8 years of school, 218 (5.93%) had completed 8 to 11 years of school, 670 (18.22%) had completed 12 years or high school, 806 (21.92%) had some college, 284 (7.72%) had post-high school training other than college, 889 (24.18%) were college graduates, 569 (15.48%) had postgraduate schooling, and 151 (4.11%) did not specify.

**Table 1:** Survey questions.

Construct	Survey Question
Trust in internet health information	In general, how much would you trust information about cancer from [the internet]? (*Not at all*, *A little*, *Some*, *A lot*)
Information security beliefs	How confident are you that safeguards (including the use of technology) are in place to protect your medical records from being seen by people who aren’t permitted to see them? Having safeguards (including the use of technology) in place has to do with the security of your medical records. (*Very confident*, *Somewhat confident*, *Not confident*)
Privacy concerns	If your medical information is sent electronically from one health care provider to another, how concerned are you that an unauthorized person would see it? Electronically means from computer to computer, instead of by telephone, mail, or fax machine. (*Very concerned*, *Somewhat concerned*, *Not concerned*)
Support for electronic medical record	Please indicate how important it is that [Doctors and other healthcare providers should be able to share your medical information with each other electronically]. (*Very important*, *Somewhat important*, *Not at all important*)
Patient access to health records	How many times did you access your personal health information online through a secure website or app in the last 12 months? (*None*, *1 to 2 times*, *3 to 5 times*, *6 to 9 times*, *10 or more times*)
Patient care quality	Overall, how would you rate the quality of healthcare you received in the past 12 months? (*Excellent*, *Very good*, *Good*, *Fair*, *Poor*)

## Results

We tested all the hypotheses in one model (as shown in Figure 2). We used R software [51] and the Lavaan package [52] to create the SEM to analyze the hypotheses. We performed checks of goodness-of-fit by checking for a nonsignificant chi-square and a low root mean square error of approximation (RMSEA)–a measure of model fit. Our chi-square test was significant (<.001) indicating other relationships among our constructs not modeled. The RMSEA was low (.099), indicating that our model fits the data. We also verified that there were no multicollinearity issues in two ways. First, we verified that all correlations were below .80 (they ranged from −0.28 to 0.35) [53]. Second, we ran a regression of all the constructs to predict perceived care quality and checked the variance inflation factors (VIF) as well [54,55]. All VIF values were close to 1 (with a range 1.03-1.12) showing no signs of multicollinearity.

**Figure 3:**
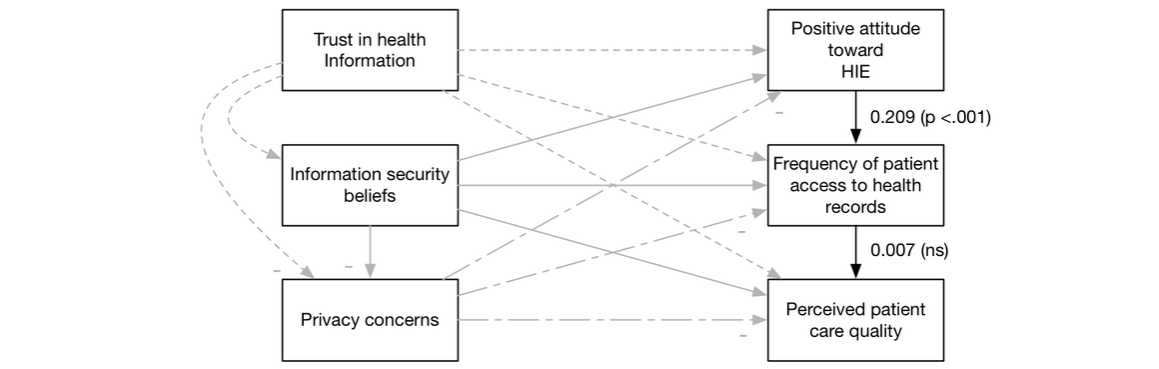
Results of H1 and H2. HIE: health information exchange.

**Figure 4:**
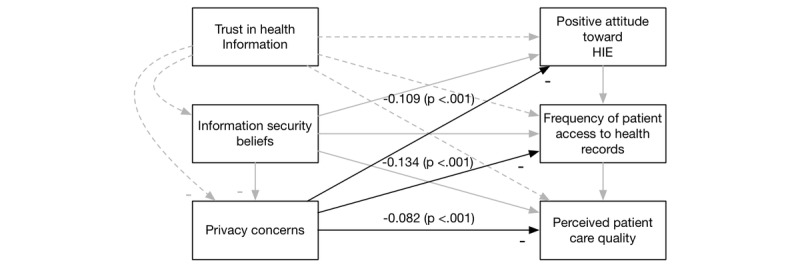
Results of H3. HIE: health information exchange.

**Figure 5:**
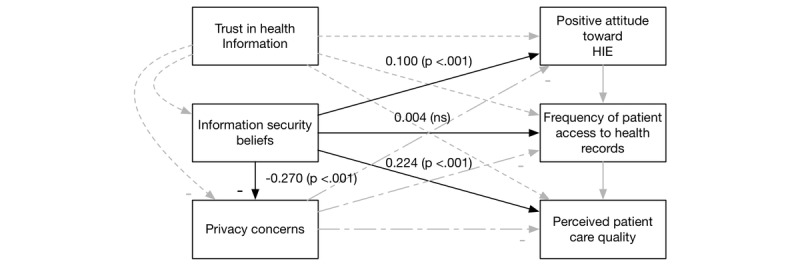
Results of H4. HIE: health information exchange.

**Figure 6:**
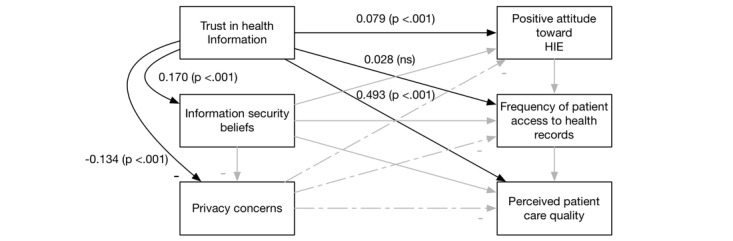
Results of H5. HIE: health information exchange.

Overall, our extension of the DMISSM to the health care system was supported. Figures 3-6 diagram the tested relationships a portion at a time to make interpreting the results easier. Most of the hypotheses were supported (see Table 2).

**Table 2:** Summary of hypothesis testing. HIE: health information exchange.

Hypothesis		Supported?
H1. Patient access to health records increases the level of perceived patient care quality.		No
H2. Positive attitudes toward HIE increase the patient’s access to health records.		Yes
**H3. Privacy concerns will**		
	(1) decrease support for electronic medical records	Yes
	(2) decrease use of patient access to health records	Yes
	(3) decrease the level of perceived patient care quality	Yes
**H4. Information security beliefs will**		
	(1) increase support for electronic medical records	Yes
	(2) increase patient access to health records	No
	(3) increase the level of perceived patient care quality.	Yes
	(4) decrease privacy concerns	Yes
**H5. Trust in internet health information will**		
	(1) increase support for electronic medical records	Yes
	(2) increase patient access to health records	No
	(3) increase the level of perceived patient care quality	Yes
	(4) increase information security beliefs	Yes
	(5) decrease privacy concerns	Yes

## Discussion

### Principal Findings

The results indicate that increased privacy concerns reduce the frequency of EMR use, positive attitudes toward HIE, and ultimately, perceptions of patient care quality. These findings confirm and extend previous reports of privacy concerns deterring patients’ adoption of HITs [6]. We also found that information security beliefs increase positive attitudes toward HIE and perceptions of patient care quality. There is, however, an indirect relationship between information security beliefs and frequency of EMR use through decreasing privacy concerns. Likewise, trust in health information has a positive association with positive attitudes toward HIE and patient care quality. Finally, the results show that patients’ positive attitudes toward HIE have a positive relationship with patient care quality.

These findings have several practical implications for health care providers and policy makers. First, it was shown that while patients’ privacy concerns impede their use of HIS and increase the negative attitude toward HIE and care quality, the perceived effectiveness of security controls lessens privacy concerns. As such, health care providers can mitigate privacy and security concerns by developing more secure privacy safeguards to prevent security attacks and unauthorized access to information. Providers must be transparent with patients [56] by providing clear information about how the security and privacy of patient data are preserved, under what circumstances data is shared, and with whom. This level of transparency, combined with adequate communication may reduce patients’ privacy concerns and reluctance to share information, consequently increasing HIT adoption and patient care quality. Second, because the trust of health information is of critical importance to the success of HIT [8], more attention needs to be paid to solutions for increasing patients’ trust. Incorrect information not only limits the efficacy of HIT and reduces patients’ trust but also negatively impacts medical decisions [57]. More research is needed to identify the antecedents of patient trust of health information in the context of HIT quality. Third, the results suggest that patients consider information sharing among health care providers to be a valuable capability in care delivery. In other words, patients believe that they benefit when their doctors have the capability to easily exchange information [58]. Entities within a health care organization are therefore encouraged to pursue solutions for effective health information exchange. A key finding is that trust in health information had no direct effect on EMR use; however, it has an indirect relationship through information privacy concerns. This shows that trust in health information lowers patients’ privacy concerns, which in turn, increases EMR use by patients.

Our research makes the following theoretical contributions: we extend the DMISSM to a health care system level, above and beyond looking at one particular technology. We also extend the model to the information assurance discipline by adding 2 new constructs of system quality: information trust and information security beliefs.

### Limitations

There are several limitations that present an opportunity for future study. First, the hypothesized model was tested using secondary data from a national survey of cancer patients. This limits our findings because the opinions expressed may not reflect those of patients suffering from other diseases as suggested in Zhang et al, 2012 [59]. More research is needed to extend our findings to other patient populations. The second limitation relates to our measurement of patient care quality; we used patients’ perception of patient care quality instead of an objective measure. While this is a limitation, the only way to get close to capturing care quality objectively is to look at readmittance data.

### Conclusions

In today’s health care organizations, information technologies have become critical to the provision of medical care. Meaningful use regulations, combined with the desire to improve care coordination, reduce costs, and improve patient engagement all depend on the success of health care technologies. Yet, there is currently limited understanding of the antecedents of HIS success. Using the Delone and Mclean Information Systems Mode l [37], we investigated the relationships between HIT quality measures and HIT use and the impact of HIT use on patient care quality. The HIT quality measures we studied included trust in health information, information security beliefs, and information privacy controls. We found strong support for the relationships between information trust and the positive attitudes toward HIE among providers, and information trust and frequency of EMR use by patients. The results also showed that trust in health information and information security beliefs have a positive relationship with patient care quality, while privacy concerns reduced patient care quality. The findings have several theoretical and practical implications. It informs health care providers and leaders of the critical importance of effective security controls. While it is known that patients care about privacy, this research shows that patients care about HIT security controls as well. Hospitals can, therefore, improve patient care by implementing effective security and privacy controls. In addition, health care providers must not only provide access to medical records but also encourage patients to check medical records frequently.

## Abbreviations

DMISSM: DeLone and McLean Information Systems Success Model
EMR: electronic medical record
HIE: health information exchange
HINTS: Health Information National Trends Survey
HIT: health information technology
PHR: patient health record
RMSEA: root mean square error of approximation
SEM: structural equation modeling
